# Factors Associated With the Discontinuation of Two Short-Course Tuberculosis Preventive Therapies in Programmatic Settings in the United States

**DOI:** 10.1093/ofid/ofae313

**Published:** 2024-06-06

**Authors:** Michael Asare-Baah, LaTweika A T Salmon-Trejo, Thara Venkatappa, Richard S Garfein, Kaylynn Aiona, Michelle Haas, Marie Nancy Séraphin

**Affiliations:** Department of Epidemiology, University of Florida, Gainsville, Florida, USA; Emerging Pathogens Institute, University of Florida, Gainsville, Florida, USA; Department of Medicine, Division of Infectious Diseases and Global Medicine, University of Florida, Gainsville, Florida, USA; Institute of Public Health, Florida A & M University, Tallahassee, Florida, USA; Division of Tuberculosis Elimination, Centers for Disease Control and Prevention, Atlanta, Georgia, USA; Division of Tuberculosis Elimination, Centers for Disease Control and Prevention, Atlanta, Georgia, USA; Herbert Wertheim School of Public Health, University of California, San Diego, California, USA; Public Health Institute at Denver Health, Denver, Colorado, USA; Division of Mycobacterial and Respiratory Infections, National Jewish Health, Denver, Colorado, USA; Emerging Pathogens Institute, University of Florida, Gainsville, Florida, USA; Department of Medicine, Division of Infectious Diseases and Global Medicine, University of Florida, Gainsville, Florida, USA

**Keywords:** latent tuberculosis infection, preventive therapy, TB treatment, treatment adherence, tuberculosis

## Abstract

**Background:**

The objective of this study was to investigate timing and risk factors for discontinuation of short-course tuberculosis preventive therapy (TPT) comparing directly observed 3-month isoniazid/rifapentine (3HP) vs self-administered 4-month rifampin (4R).

**Methods:**

This was a subanalysis of a 6-month health department cohort (2016–2017) of 993 latent tuberculosis infection (LTBI) patients initiating 3HP (20%) or 4R (80%). Time at risk of TPT discontinuation was compared across regimens. Risk factors were assessed using mixed-effects Cox models.

**Results:**

Short-course TPT discontinuation was higher with 4R (31% vs 14%; *P* < .0001), though discontinuation timing was similar. Latino ethnicity (hazard ratio [HR], 1.80; 95% CI, 1.20–2.90) and adverse events (HR, 4.30; 95% CI, 2.60–7.30) increased 3HP discontinuation risk. Social–behavioral factors such as substance misuse (HR, 12.00; 95% CI, 2.20–69.00) and congregate living (HR, 21.00; 95% CI, 1.20–360.00) increased 4R discontinuation risk.

**Conclusions:**

TPT discontinuation differed by regimen, with distinct risk factors. Addressing social determinants of health within TPT programs is critical to enhance completion rates and reduce TB disease risk in marginalized populations.

Although substantial progress has been made in curtailing tuberculosis (TB) in the United States, the annual decline in incidence rate is insufficient to meet the elimination goal of 1 case per million population [1]. Approximately 8000 TB cases were reported in the United States in 2022, with a prevalence of 2.5 cases per 100 000 persons [2]. Surveillance data suggest that 80% of these cases are due to long-standing latent tuberculosis infection (LTBI) that progresses to TB disease [1]. Infectious TB cases present an exposure risk for their close household and social contacts, some of whom may become latently infected or develop primary TB disease [1, 3]. An estimated 12–14 million people in the United States have LTBI [4, 5]. To advance toward TB elimination, we must ensure access to care and TB preventive therapy (TPT) for individuals with LTBI to prevent them from progressing to TB disease [6].

The first approved LTBI treatment regimen required 6–12 months of a daily dose of isoniazid (INH) [6]. While the therapy is effective, patient adherence and treatment completion rates have been low due to medication side effects and treatment fatigue [7]. Substantial progress in designing shorter, more tolerable LTBI regimens in recent years has increased TPT completion rates [8, 9]. Currently, we can treat people with LTBI in 3–4 months with either a weekly course of a combination of INH and rifapentine (3HP) or daily rifampin for 4 months (4R) [9–11]. In people with HIV (PWH), the clinical guidelines allow using 4R to treat LTBI in combination with a tenofovir disoproxil fumarate–containing antiretroviral therapy (ART) regimen [12]. The regimen can also be used to treat LTBI in patients who are either not on ART or receiving dolutegravir or efavirenz [13]. Among adults, TPT completion rates with these short-course LTBI regimens hover around 87% when treated with 3HP [8–10, 14] and 70% with 4R [8]. A new 1-month regimen of INH and rifapentine, which is noninferior to 9-month INH among PWH, is expected to have even higher completion rates [15].

Although shorter, more tolerable LTBI treatment regimens have increased rates of completion, completion remains suboptimal, raising interest in investigating factors that predict noncompletion. Problems with INH completion rates have been well described; however, little is known about the factors associated with failing to complete 3HP and 4R. For example, in previous work among LTBI patients treated with 6–9 months of daily INH in health department clinics, we have seen that 49% of patients discontinued therapy [16]. Factors found to predict INH noncompletion include patient difficulties with transportation and lost income from time off needed for clinic visits [17]. In analyses investigating the association between the duration of US residency or other risk factor proxies of progression to TB disease, such as comorbid disease states, alcohol use, or younger age, we have seen that treatment duration alone does not fully explain the decision of patients to discontinue LTBI therapy [18]. There is a need to understand the risk factors associated with time to treatment discontinuation among LTBI patients offered 3HP or 4R to inform strategies to maximize treatment completion rates with these shorter regimens.

We investigated the timing of LTBI treatment discontinuation and described its association with social, demographic, and clinical risk factors that increase the risk of progression to TB disease among health department patients treated with 3HP or 4R. We also investigated whether the factors associated with LTBI treatment discontinuation differ by treatment regimen (3HP or 4R).

## METHODS

### Patient Consent

This study was reviewed at the Centers for Disease Control and Prevention (CDC) and excluded from institutional review board (IRB) review as research not involving identifiable human subjects. Some individual study sites relied on the CDC determination. It was additionally reviewed and approved by the IRBs of Johns Hopkins University School of Medicine, University of Maryland, Maryland Department of Health, North Texas Regional IRB, and Atrium Health.

### Study Setting

We conducted a subanalysis of data collected as part of an observational cohort study in collaboration with 15 health department TB clinics in 10 states across the United States [19]. The primary study objective was to describe the attrition rate at each step of the TB prevention cascade, starting with LTBI screening through treatment completion within partner public health clinics [19]. Extensive data on demographics, risk factors, screening, and treatment outcomes were abstracted from clinic records for patients who visited a participating public health TB clinic during a 6-month cohort period. Each clinic had a different 6-month time frame for their cohort. Data collection began in January 2016. Eight study sites collected TPT outcome data retrospectively (January 2016–October 2017) for their cohorts, while the other 7 sites prospectively (January 2017–October 2017) followed patients initiating TPT for their outcomes.

### Study Population

All patients diagnosed with LTBI who were offered and initiated TPT were eligible for this study. We included patients with LTBI in the analysis if they initiated TPT with 3HP or 4R and had at least 1 follow-up visit on record. Patients with LTBI were excluded if they discontinued therapy because they were subsequently diagnosed with TB disease.

### Measures

#### Treatment Outcomes Definition

TPT outcomes were recorded as complete or not complete, a classification that captured both the time spent in therapy and the number of doses ingested [20]. For example, a patient treated with 3HP would complete TPT after ingesting 11 doses within 16 weeks of therapy initiation, while a patient treated with 4R would need to ingest 120 doses within 6 months to complete TPT [20]. The treatment start date, the clinic discharge date, the reason for closing the treatment plan, and the treatment completion status were available for all patients. Patients who interrupted therapy and returned after <6 weeks were continued on the same regimen. However, absences >6 weeks required the patient to restart the treatment regimen. For these patients, the outcome of the first TPT regimen was considered incomplete in our analysis.

TPT treatment administration was documented as self-administered therapy (SAT) or directly observed therapy (DOT) for most LTBI patients. Those who received video DOT (VDOT) were classified as DOT, and children who had their treatment administered by a parent at home were classified as SAT. For patients who did not complete the prescribed therapy, the reason for noncompletion was documented as lost to care, adverse events/chose to stop, or died (unrelated to treatment). We reclassified patients who did not complete treatment as lost to care if a reason for noncompletion was unavailable in the medical record.

#### Treatment Follow-up and Censoring

We treated adverse events/chose to stop, died (unrelated to treatment), and treatment completed as observed censored events. We calculated the weeks at risk of treatment discontinuation using the treatment start date and the respective observed treatment interruption event date. In cases where treatment was completed but the date was missing, we used the discharge date to calculate the period in weeks at risk of treatment interruption. For patients lost to care and for whom no follow-up visits occurred, their time at risk for discontinuation was considered 0. Otherwise, we used the discharge date, which is an administrative closure of the treatment plan triggered after 2 consecutive missed treatment visits.

#### Demographic Factors

The demographic factors of interest included sex at birth, race, ethnicity, and age at the first visit, categorized as 0–24, 25–44, 45–64, and ≥65 years. We grouped patients by self-reported race/ethnicity: Hispanic/Latino or non-Hispanic White, Black, Asian, or other. We classified Native Americans, Pacific Islanders, and those with missing race as other due to small numbers and to preserve privacy. Birth origin was categorized as US-born or non-US-born. For individuals born outside the United States, we calculated the number of years lived in the United States using their US arrival date. We combined information on country of birth and the number of years lived in the United States to categorize the sample into US-born, non-US-born/≤5 years, and non-US-born/>5 years.

#### Medical, Social, and Occupational Risk Factors

The primary reason for LTBI evaluation was categorized as TB contact investigation, screening, or administrative (other), which included TB infection screening for work or school clearance purposes. Screening encompassed TB infection testing and treatment in immigrants, refugees, and other high-risk groups. We grouped the study population into those who had either a tuberculin skin test (TST) only, an interferon gamma release assay (IGRA) only, or both a TST and IGRA. The occupational risk factors for TB infection modeled included working in corrections or as a health care worker. LTBI patients age <18 years were categorized in the no-risk group for occupation. Medical risk factors for LTBI progression that we evaluated included diabetes, HIV, hepatitis, and non-HIV immunosuppressive conditions. Patients with LTBI were classified as having a medical risk factor for TB disease if they had 1 or more of these diagnoses. The self-reported social risk factors for LTBI included homelessness, alcohol use disorder, substance use disorder, which included injection and noninjection drug use, and residence in a long-term care or corrections facility in the year before screening. As there were too few observations to model each risk factor separately, we grouped alcohol use disorder and substance use to create 1 indicator of social risk factors for TB infection. We classified those reporting experiencing homelessness in the year before LTBI diagnosis or who were residents in a correctional or long-term care facility separately as having a congregate risk for TB infection.

### Statistical Analysis

We estimated the probability of treatment discontinuation over time stratified by demographic and risk factors of interest and displayed the results using Kaplan-Meier curves. We compared the time to treatment discontinuation within levels of categorical covariates using log-rank with log–log transformation to estimate confidence intervals. We fitted bivariate and fully saturated multivariable mixed-effect Cox proportional hazards models to the hazard ratio (HR) with 95% CI for the individual contribution of each risk factor of interest to TPT discontinuation [21]. The study site locations (East, Pacific, South, and West) were included in the models to control for potential regional differences in LTBI treatment completion rates. To ensure the validity of the subsequent multivariate analysis, the proportional hazard (PH) assumptions were verified by visually inspecting the group-based empirical cumulative hazard curves for potential violations of proportionality. We applied Efron correction to resolve ties in the treatment disruption times.

## RESULTS

### Study Population and Characteristics

**Figure 1.**
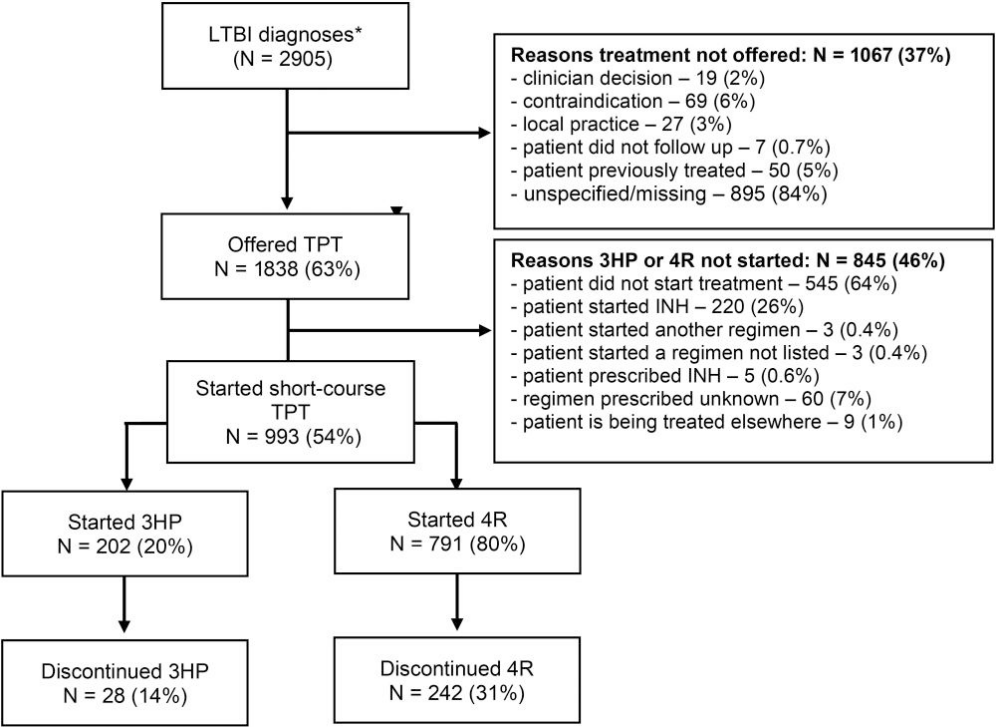
Flow diagram illustrating selection of the study population to investigate timing and risk factors for discontinuing TPT with 3HP or 4R. Eligible participants are those diagnosed with LTBI who initiated therapy with 3HP or 4R. *Those with ATS classification code = 2 (LTBI, no disease) and chest x-ray results. Proportions are rounded to the nearest whole number. Abbreviations: 3HP, 3-month weekly dose of isoniazid and rifapentine; 4R, 4-month daily dose of rifampin; ATS, American Thoracic Society; INH, isoniazid; LTBI, latent tuberculosis infection; TPT, tuberculosis preventive therapy.

Of the 2905 patients diagnosed with LTBI, 63% (1838/2905) were offered TPT, and 54% (993/1838) of those offered treatment started short-course TPT. Of the 993 patients who elected to start short-course TPT, 80% (791/993) were treated with the 4R regimen by self-administration, while 20% (202/993) received 3HP exclusively by DOT (Figure 1).

Of the 993 LTBI patients who started a short-term regimen, 51% were male. The racial and ethnic composition of the study sample was 30% Hispanic/Latino, 26% Asian, 31% Black, 10% White, and 3% other. Ninety-three percent of LTBI patients in our sample were born outside of the United States, and of those, 71% (702/993) had resided in the United States for ≤5 years before LTBI evaluation. Two-thirds of the sample had IGRA-only testing for LTBI screening, while 15% received both a TST and IGRA. Screening for immigration, refugee, and other high-risk groups was the main reason for LTBI evaluation (67%). The other reasons were administrative clearance for work or school (17%) and contact investigation (15%). The prevalence of medical (10%), social (3%), congregate (2%), or occupational (4%) risk factors for TB infection or progression to TB disease were low (Table 1).

**Table 1.** Study Population Characteristics Stratified by Short-Course Tuberculosis Preventive Treatment Outcomes

	Total Population, No. (%)	Treatment Completed, No. (%)	Lost to Care, No. (%)	Adverse Event or Chose to Stop, No. (%)
Total sample	993	723	165	104
Sex at birth = male	508 (51.0)	357 (49.0)	98 (59.0)	52 (50.0)
Self-reported race/ethnicity				
Hispanic/Latino ethnicity	294 (30.0)	171 (24.0)	65 (39.0)	58 (56.0)
Non-Hispanic Asian	262 (26.0)	227 (31.0)	23 (14.0)	12 (12.0)
Non-Hispanic Black	311 (31.0)	229 (32.0)	60 (36.0)	22 (21.0)
Non-Hispanic White	99 (10.0)	72 (10.0)	15 (9.0)	11 (11.0)
Non-Hispanic other	27 (3.0)	24 (3.0)	2 (1.0)	1 (1.0)
Age group				
0–24 y	204 (20.0)	155 (21.0)	34 (21.0)	15 (14.0)
25–44 y	477 (48.0)	349 (48.0)	82 (50.0)	45 (43.0)
45–64 y	259 (26.0)	186 (26.0)	41 (25.0)	32 (31.0)
≥65 y	53 (5.0)	33 (5.0)	8 (5.0)	12 (11.0)
Birth origin				
US-born	66 (7.0)	40 (5.0)	19 (12.0)	7 (7.0)
Non-US-born, ≤5 y	702 (71.0)	527 (73.0)	106 (64.0)	68 (65.0)
Non-US-born, >5 y	216 (22.0)	148 (20.0)	39 (24.0)	29 (28.0)
Missing or unknown	9 (1.0)	8 (1.0)	1 (1.0)	0 (0.0)
LTBI screening test				
TST	183 (18.0)	126 (17.0)	33 (20.0)	24 (23.0)
IGRA	660 (66.0)	464 (64.0)	121 (73.0)	74 (71.0)
TST & IGRA	150 (15.0)	133 (18.0)	11 (7.0)	6 (6.0)
Primary evaluation reason				
Contact investigation	153 (15.0)	112 (15.0)	27 (16.0)	14 (13.0)
Screening	667 (67.0)	490 (68.0)	113 (68.0)	63 (60.6)
Other	173 (17.0)	121 (17.0)	25 (15.0)	27 (26.0)
Medical risks^a^				
No	592 (60.0)	443 (61.0)	90 (54.0)	59 (57.0)
Yes	97 (10.0)	68 (9.0)	20 (12.0)	9 (9.0)
Unknown	304 (31.0)	212 (29.0)	55 (33.0)	36 (35.0)
Social risks^b^				
No	825 (83.0)	596 (82.0)	129 (79.0)	99 (95.0)
Yes	25 (3.0)	19 (3.0)	5 (3.0)	1 (1.0)
Unknown	141 (14.0)	107 (15.0)	30 (18.0)	4 (4.0)
Congregate risks^c^				
No	691 (70.0)	502 (69.0)	103 (63.0)	86 (83.0)
Yes	20 (2.0)	11 (2.0)	7 (4.0)	2 (2.0)
Unknown	280 (28.0)	209 (29.0)	54 (33.0)	16 (15.0)
Occupational risks^d^				
No	567 (57.0)	405 (56.0)	82 (50.0)	80 (77.0)
Yes	41 (4.0)	27 (4.0)	7 (4.0)	7 (7.0)
Unknown	383 (39.0)	290 (40.0)	75 (46.0)	17 (16.0)
Treatment management				
Directly observed therapy	160 (16.0)	142 (20.0)	12 (7.0)	5 (5.0)
Self-administered therapy	731 (74.0)	502 (69.0)	136 (82.0)	93 (89.0)
Unknown/missing	102 (10.0)	79 (11.0)	17 (10.0)	6 (6.0)
Study site				
East	564 (57.0)	398 (55.0)	88 (53.0)	78 (75.0)
Pacific	41 (4.0)	37 (5.0)	1 (1.0)	3 (3.0)
South	237 (24.0)	171 (24.0)	46 (28.0)	19 (18.0)
West	151 (15.0)	117 (16.0)	30 (18.0)	4 (4.0)

One patient included in the total population who died due to causes unrelated to treatment is not shown in the table. Proportions are rounded to the nearest whole number and may therefore sum to >100%.

Abbreviations: IGRA, interferon gamma release assay; LTBI, latent tuberculosis infection; TST, tuberculin skin test.

^a^Includes diabetes, HIV, hepatitis, and other immunosuppressive conditions.

^b^Includes excess alcohol and recreational drug (injection and noninjection) use.

^c^Includes homeless, corrections, and long-term care facility residents.

^d^Includes employment in corrections or as a health care worker.

The majority of study participants (791 out of 993 with LTBI) were predominantly treated with the 4R regimen. The choice of regimen did not differ by sex at birth, birth origin, the primary reason for LTBI evaluation, or patients’ medical and congregate risks for TB infection or TB disease (Table 2). Patients started on 4R differed significantly from patients started on 3HP on self-reported race/ethnicity, age at LTBI diagnosis, and their social and occupational risk factors for TB infection and TB disease. The choice of treatment regimen also significantly differed by study site region and whether the patient was screened for TB infection using TST, IGRA, or both TST and IGRA testing (Table 2**)**.

**Table 2.** Study Population Characteristics Stratified by Choice of Short-Course Tuberculosis Preventive Treatment Regimen Started

	Total Population, No. (%)	3HP Regimen, No. (%)	4R Regimen, No. (%)	*P* Value*
Total sample	993	202	791	
Sex at birth = male	508 (51.0)	105 (52.0)	403 (51.0)	.855
Self-reported race/ethnicity	…	…	…	.009
Hispanic/Latino ethnicity	294 (30.0)	43 (21.0)	251 (32.0)	
Non-Hispanic Asian	262 (26.0)	50 (25.0)	212 (27.0)	
Non-Hispanic Black	311 (31.0)	78 (39.0)	233 (29.0)	
Non-Hispanic White	99 (10.0)	22 (11.0)	77 (10.0)	
Non-Hispanic other	27 (3.0)	9 (4.0)	18 (2.0)	
Age group	…	…	…	.028
0–24 y	204 (20.0)	46 (23.0)	158 (20.0)	
25–44 y	477 (48.0)	109 (54.0)	368 (46.0)	
45–64 y	259 (26.0)	42 (21.0)	217 (27.0)	
≥65 y	53 (5.0)	5 (2.0)	48 (6.0)	
Birth origin	…	…	…	.766
US-born	66 (7.0)	12 (6.0)	54 (7.0)	
Non-US-born, ≤5 y	702 (71.0)	148 (73.0)	554 (70.0)	
Non-US-born, >5 y	216 (22.0)	41 (20.0)	175 (22.0)	
Missing or unknown	9 (1.0)	1 (0.0)	8 (1.0)	
LTBI screening test	…	…	…	<.001
TST	183 (18.0)	16 (8.0)	167 (21.0)	
IGRA	660 (66.0)	140 (69.0)	520 (66.0)	
TST & IGRA	150 (15.0)	46 (23.0)	104 (13.0)	
Primary evaluation reason	…	…	…	.384
Contact investigation	153 (15.0)	30 (15.0)	123 (15.0)	
Screening	667 (67.0)	143 (71.0)	524 (66.0)	
Other	173 (17.4)	29 (14.0)	144 (18.0)	
Medical risks^a^	…	…	…	.099
No	592 (59.6)	112 (55.0)	480 (61.0)	
Yes	97 (9.8)	16 (8.0)	81 (10.0)	
Unknown	304 (30.6)	74 (37.0)	230 (29.0)	
Social risks^b^	…	…	…	.014
No	825 (83.2)	153 (76.0)	672 (85.0)	
Yes	25 (2.5)	6 (3.0)	19 (2.0)	
Unknown	141 (14.2)	41 (20.0)	100 (13.0)	
Congregate risks^c^	…	…	…	.746
No	691 (69.7)	143 (71.0)	548 (69.0)	
Yes	20 (2.0)	3 (1.0)	17 (2.0)	
Unknown	280 (28.3)	54 (27.0)	226 (29.0)	
Occupational risks^d^	…	…	…	.002
No	567 (57.2)	106 (53.0)	461 (58.0)	
Yes	41 (4.1)	1 (0.0)	40 (5.0)	
Unknown	383 (38.6)	93 (46.0)	290 (37.0)	
Study site	…	…	…	<.001
East	564 (56.8)	92 (45.0)	472 (60.0)	
Pacific	41 (4.1)	3 (1.0)	38 (5.0)	
South	237 (23.9)	65 (32.0)	172 (22.0)	
West	151 (15.2)	42 (21.0)	109 (14.0)	
Treatment outcome	…	…	…	<.001
Treatment completed	723 (73.0)	174 (86.0)	549 (69.0)	
Lost to care	165 (17.0)	21 (10.0)	144 (18.0)	
Adverse event or chose to stop	104 (10.0)	6 (3.0)	98 (12.0)	

Proportions are whole numbers. For *P* value, yes vs all other levels.

Abbreviations: 3HP, 3-month weekly dose of isoniazid and rifapentine by direct observation; 4R, 4-month daily dose of rifampin by self-administration; IGRA, interferon gamma release assay; LTBI, latent tuberculosis infection; TPT, tuberculosis preventive therapy; TST, tuberculin skin test.

^*^ *P* value <.05 to assess the statistical association between patient population characteristics and prescribed treatment regimen. Fisher exact tests were computed when the expected cell count was <5. Otherwise, chi-square tests were used.

^a^Includes diabetes, HIV, hepatitis, and other immunosuppressive conditions.

^b^Includes excess alcohol use, injection, noninjection, and recreational drug use.

^c^Includes homeless, corrections, and long-term care facility residents.

^d^Includes employment in corrections or as a health care worker.

### Timing of Short-Course Tuberculosis Preventive Therapy Discontinuation

**Figure 2.**
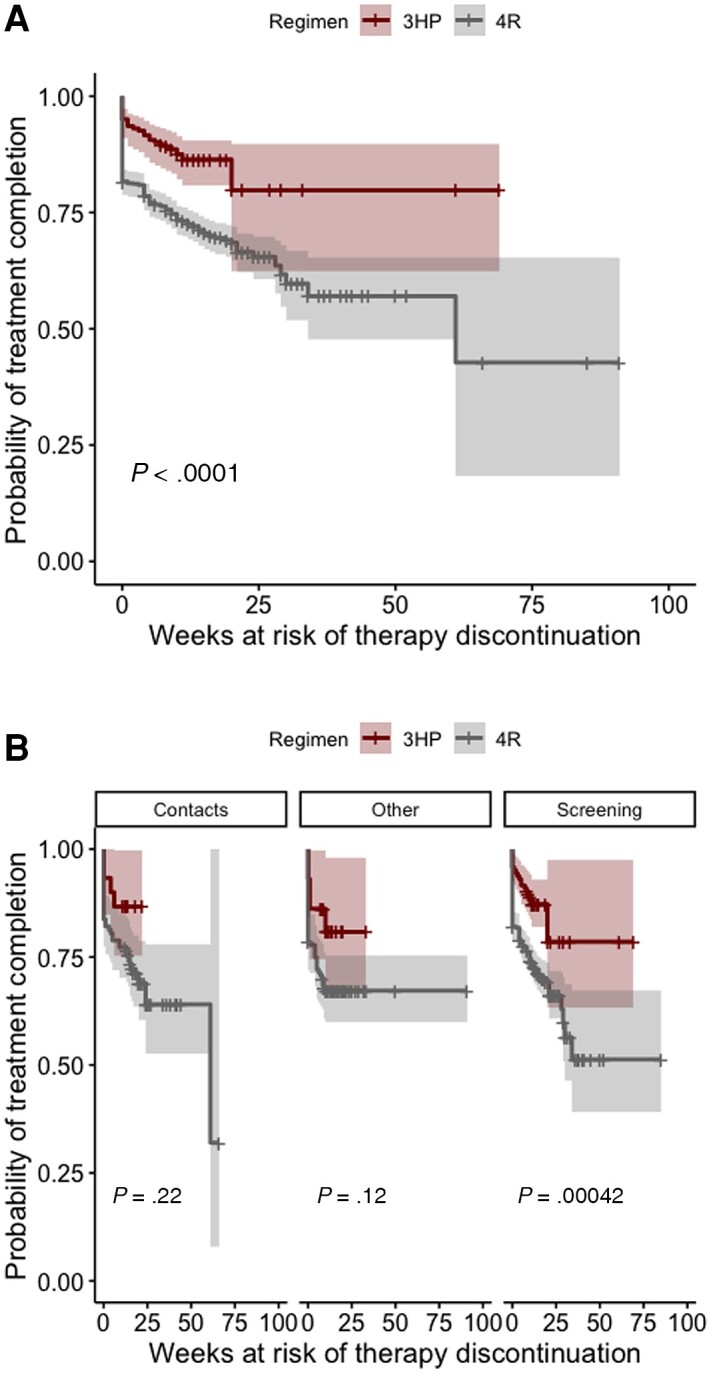
Kaplan-Meier estimates of the probability of short-course tuberculosis preventive therapy completion among patients with LTBI treated with a 3HP or 4R regimen at 15 health department TB clinics in the United States. Figure panels show the log-rank test *P* value for the hypothesis of equal probabilities of completing either regimen overall (*A*) and for each of the 3 main reasons health department patients were evaluated for LTBI (*B*). A Welch 2-sample *t* test comparing the mean treatment duration before interruption suggests no significant difference in the number of weeks spent in therapy between the 2 treatment groups (*t* = 0.12; 95% CI, −2.0 to 2.3 weeks) before treatment discontinuation. Abbreviations: 3HP, 3-month weekly dose of isoniazid and rifapentine; 4R, 4-month daily dose of rifampin; LTBI, latent tuberculosis infection; TB, tuberculosis.

The risk of short-course TPT discontinuation was 27% overall and was significantly higher among patients receiving 4R compared with 3HP (31% vs 14%; *P* < .0001). As the treatment completion rate in both groups was high and we right-censored patients who completed therapy, the Kaplan-Meier analysis could not determine median survival times. On average, patients discontinued short-course TPT within 4 weeks of treatment initiation, and the mean timing of treatment interruption did not differ significantly between treatment groups (t = 0.12; 95% CI, −2.00 to 2.30 weeks). The probability of completing either regimen was similar among contacts and other high-risk groups. However, it differed significantly between patients with LTBI who were evaluated primarily as part of refugee, immigration, and other targeted screening efforts (Figure 2).

### Risk Factors for Short-Course Tuberculosis Preventive Therapy Discontinuation

Patients who discontinued the 3HP or 4R regimen displayed no significant differences from those who completed therapy across several factors evaluated, including primary LTBI evaluation reason, LTBI screening test, and medical risk factors for progression to TB disease (Table 3). LTBI patients were at increased risk of discontinuing TPT with 3HP if they were Latino (HR, 1.80; 95% CI, 1.20–2.90) or experienced an adverse event during therapy (HR, 4.30; 95% CI, 2.60–7.30). Male sex at birth (HR, 1.30; 95% CI, 1.00–1.70) was associated with a 30% higher risk of TPT discontinuation, although the confidence interval for the point estimate suggested that the risk is not significantly different from that of female sex at birth. On the other hand, non-US born LTBI patients were less likely to discontinue 3HP (HR, 0.59; 95% CI, 0.36–0.98). LTBI patients treated with 4R were more likely to discontinue 4R if they self-identified as White (HR, 5.00; 95% CI, 1.10–22.00), were experiencing substance misuse (HR, 12.00; 95% CI, 2.20–69.00), or had a history of homelessness or incarceration with a congregate risk (HR, 21.00; 95% CI, 1.20–360.00) for TB infection. Age was also a significant factor, as patients aged 25–44 and 45–64 years were less likely to discontinue 4R than those aged 0–24 years, respectively (HR, 0.26; 95% CI, 0.09–0.75; and HR, 0.11; 95% CI, 0.02–0.65). Based on the multivariate analysis adjusted for TB disease risk factors, no other risk factors evaluated significantly influenced the risk of treatment discontinuation (Table 3).

**Table 3.** Risk Factors for the Discontinuation of Short-Course Tuberculosis Preventive Therapy With 3HP or 4R Among Health Department Patients

Characteristics	Bivariate HR for 3HP Discontinuation, HR (95% CI)	Multivariate HR for 3HP Discontinuation, HR (95% CI)	Bivariate HR for 4R Discontinuation, HR (95% CI)	Multivariate HR for 4R Discontinuation, HR (95% CI)
Population at risk	202	202	791	791
No. of discontinuation events	28	28	242	242
Sex at birth = male	1.30 (0.99–1.70)	1.30 (1.00–1.70)	1.90 (0.87–4.30)	1.20 (0.48–3.00)
Self-reported race/ethnicity				
Non-Hispanic Asian	Ref	Ref	Ref	Ref
Non-Hispanic Black	1.60 (0.98–2.50)	1.50 (0.95–2.50)	0.76 (0.23–2.60)	0.37 (0.08–1.80)
Non-Hispanic White	1.50 (0.95–2.50)	1.00 (0.60–1.80)	*3.30 (1.10–10.00)*	*5.0 (1.10–22.00)*
Non-Hispanic other	1.10 (0.61–2.10)	0.69 (0.33–1.40)	0.32 (0.05–1.90)	0.36 (0.03–4.10)
Hispanic/Latino ethnicity	1.20 (0.87–1.60)	*1.80 (1.20–2.90)*	0.74 (0.28–1.90)	0.76 (0.13–4.30)
Age group				
0–24 y	Ref	Ref	Ref	Ref
25–44 y	1.30 (0.91–1.90)	1.40 (0.93–2.00)	*0.42 (0.19–0.96)*	*0.26 (0.09–0.75)*
45–64 y	1.10 (0.74–1.60)	1.10 (0.73–1.70)	*0.17 (0.04–0.76)*	*0.11 (0.02–0.65)*
≥65 y	1.10 (0.60–1.90)	0.94 (0.51–1.80)	2.60 (0.51–13.00)	3.40 (0.37–32.00)
Birth origin				
US-born	Ref	Ref	Ref	Ref
Non-US-born	0.71 (0.45–1.10)	*0.59 (0.36–0.98)*	0.54 (0.16–1.90)	0.67 (0.08–5.30)
LTBI screening test				
TST	Ref	Ref	Ref	Ref
IGRA	0.86 (0.61–1.20)	0.75 (0.50–1.10)	1.30 (0.29–5.60)	2.90 (0.47–18.00)
TST & IGRA	0.56 (0.30–1.00)	0.54 (0.28–1.00)	0.57 (0.09–3.70)	2.90 (0.29–28.00)
Primary evaluation reason				
Contact investigation	Ref	Ref	Ref	Ref
Screening	1.10 (0.73–1.50)	1.30 (0.84–2.00)	1.30 (0.40–3.90)	1.70 (0.26–11.00)
Other	0.87 (0.56–1.40)	1.00 (0.65–1.60)	0.91 (0.20–4.20)	0.99 (0.12–8.30)
Medical risks^a^				
No	Ref	Ref	Ref	Ref
Yes	1.30 (0.85–2.00)	1.40 (0.89–2.20)	1.20 (0.26–5.30)	2.30 (0.32–17.00)
Unknown	1.30 (0.91–1.70)	1.40 (0.97–2.00)	1.70 (0.61–4.70)	2.20 (0.68–6.90)
Social risks^b^				
No	Ref	Ref	Ref	Ref
Yes	0.59 (0.19–1.80)	0.73 (0.23–2.30)	*6.00 (1.70–21.00)*	*12.00 (2.20–69.00)*
Unknown	0.81 (0.46–1.40)	0.83 (0.47–1.40)	1.50 (0.55–3.90)	1.60 (0.41–6.20)
Congregate risks^c^				
No	Ref	Ref	Ref	Ref
Yes	1.80 (0.74–4.20)	1.30 (0.52–3.30)	*9.60 (2.00–46.00)*	*21.00 (1.20–360.00)*
Unknown	1.20 (0.78–2.00)	1.30 (0.80–2.10)	*2.40 (1.10–5.20)*	*4.40 (1.50–13.00)*
Treatment adverse reaction = yes	3.40 (2.10–5.60)	4.30 (2.60–7.30)	11.00 (2.80–40.00)	3.50 (0.71–17.00)

For these analyses, we collapsed the non-US birth categories to prevent model misspecification due to empty cells. Italicized text indicates statistically significant results.

Abbreviations: 3HP, 3-month weekly dose of isoniazid and rifapentine; 4R, 4-month daily dose of rifampin; HR, hazard ratio; IGRA, interferon-gamma release assay; TST, tuberculin skin test.

^a^Includes diabetes, HIV, hepatitis, and other immunosuppressive conditions.

^b^Includes excess alcohol and recreational drug (injection and noninjection) use.

^c^Includes homelessness, corrections, and long-term care facility residents.

## DISCUSSION

We investigated the timing of LTBI treatment discontinuation and its association with demographic, social, and clinical risk factors in a large observational cohort of patients with LTBI receiving treatment with either 3HP or 4R in 15 health department TB clinics across the United States. We found that a higher proportion of patients (86%) who received 3HP treatment completed their treatment compared with those who received 4R treatment (69%), with a similar mean number of weeks (4 weeks) spent in therapy between the 2 treatment groups before treatment discontinuation. This finding is consistent with previous research [20]. Our results suggest that the time to TPT discontinuation is not different between the 2 regimens. Nevertheless, several extraneous factors not captured in our analysis likely influenced the differing treatment outcomes between the 2 groups. The shorter duration and weekly dosing of the 3HP regimen likely increased patients’ motivation to complete TPT due to the limited impact the regimen had on other aspects of the patients’ lives [9]. Also, the 3HP regimen was administered by direct observation with remote video DOT offered to some patients. DOT ensures that patients take their medication as prescribed and reduces nonadherence risk. In this study, 4R was primarily not provided via DOT. Our study showed a higher risk of treatment discontinuation in LTBI patients with a history of alcohol and recreational drug use, as captured as social risk in patients treated with 4R but not for patients treated with 3HP, presumably because 3HP was exclusively by DOT. Thus, discontinuation events among those on 3HP were mainly due to treatment-associated adverse events. While DOT ensures treatment completion, it can be burdensome for patients and public health resources [22]. To this effect, 3HP is now offered by SAT with comparable completion rates to 3HP by DOT [23]. One interpretation of our findings is that TPT discontinuation rates would be comparable in 3HP and 4R if the prevalence of social and congregate risk factors were comparable between the 2 treatment groups. Substance use disorders can negatively impact medication adherence through complications like drug–drug interactions [24]. Also, substance use often coincides with unstable housing and co-occurring medical conditions, further challenging successful TPT completion in these patients [25–27]. The complex individual and systemic barriers patients face underscore the need for integrated, harm reduction–based LTBI care models to promote completion and prevent progression to active TB disease. Experiencing homelessness and incarceration are also associated with a higher risk of TPT discontinuation [11, 28]. Our study found significantly higher TPT discontinuation among the 4R group associated with congregate settings. Unstable housing situations and disruptions in care during incarceration and release likely impede these patients’ ability to consistently access health care and adhere to treatment, increasing their risk of dropout [25, 27]. Targeted interventions like discharge planning and transitional housing assistance could help overcome the systemic barriers people face in accessing and completing LTBI therapy [25, 26, 29]. Addressing the social determinants underlying treatment discontinuation could ensure successful completion and prevent progression to active TB.

Our findings are subject to the limitations inherent to using programmatic data collected for clinical management and surveillance, not research purposes, such as missing data and unmeasured confounders. Such confounding factors may include language barriers, educational levels, and other socioeconomic or behavioral variables, which either had a high percentage of missingness or were unavailable in the data set. Additionally, the effect of a small sample size could have influenced the reliability of our estimates; hence, caution should be taken when interpreting the results. We also do not have information on why some patients were prescribed 3HP and others 4R; thus there may be some selection bias. We analyzed program data collected before the widespread adoption of 3HP; consequently, our subanalysis may capture a healthier cohort of LTBI patients if providers were more hesitant to prescribe 3HP to patients they perceived as at higher risk of treatment complications. As LTBI patients received monthly medication refills, the exact discontinuation date and details on adherence intervals were unknown in some cases. We used the administrative closure date to indicate treatment stoppage, but this may not precisely capture the actual timing of treatment interruption.

Additionally, we lacked detailed measurements on influential variables such as social support systems, current substance use behaviors, and patient–provider communications likely to influence adherence and completion. Access to more granular patient-centered information could clarify the circumstances and nuanced reasons underlying LTBI treatment discontinuation. Future research would benefit from prospectively gathering data with specific adherence indicators, patient-centered experience measures, and larger sample sizes. This would generate a richer understanding of the multilevel factors and different patient demographics influencing LTBI treatment completion status. Enhanced data validity could further strengthen the generalizability of conclusions to guide targeted interventions promoting completion, especially in the groups at highest risk. While our analysis provides initial evidence on factors associated with noncompletion, access to more robust data sources will build on these findings to deepen scientific knowledge in this critical area.

In summary, our study did not reveal a significant difference in time to TPT discontinuation for patients with LTBI treated with 3HP, defined by some programs as 11 weekly doses ingested over 16 weeks to complete therapy, compared with LTBI patients treated with 4R who needed to ingest 120 daily doses over 6 months to complete therapy [30, 31]. Nevertheless, the motivations for treatment interruption within the 2 treatment groups diverged noticeably. In analyses adjusted for demographic, clinical, and social risk factors that increase the risk of TB progression, patients treated with 3HP interrupted therapy due to treatment-associated adverse events or patient/provider decision to stop, while patients treated with 4R were more likely to discontinue therapy due to social and congregate risk factors that also impact access to care. These findings underscore the importance of considering the multifaceted demographic and social risk factors influencing TPT nonadherence and discontinuation in clinical practice. Our analysis reveals several demographic and social risk factors that could be the focus of interventions to improve TPT completion rates in people who are members of historically marginalized populations.
